# Prognostic role of *TET2* deficiency in myelodysplastic syndromes: A meta-analysis

**DOI:** 10.18632/oncotarget.17177

**Published:** 2017-04-18

**Authors:** Yun Lin, Zhijuan Lin, Kun Cheng, Zhihong Fang, Zhifeng Li, Yiming Luo, Bing Xu

**Affiliations:** ^1^ Department of Hematology, The First Affiliated Hospital of Xiamen University, Xiamen, P.R. China; ^2^ Thoracic Department, People's Hospital Affiliated to Fujian University of Traditional Chinese Medicine, Fuzhou, P.R. China

**Keywords:** myelodysplastic syndrome, *TET2* mutation, prognosis, meta-analysis

## Abstract

*Tet methylcytosine dioxygenase2 gene (TET2)* is one of the most frequently mutated gene in myeloid neoplasm, but the prognostic role of *TET2* aberrations in myelodysplastic syndromes (MDS) remains unclear. Therefore, we performed a meta-analysis. Fourteen eligible studies with 1983 patients were included in this meta-analysis. Among these, 2 studies evaluated the impact that the *TET2* expression level had on the prognosis. The combined hazard ratios (HR) estimated for overall survival (OS) was 1.00 (95%CI: 0.74 to 1.37; p=0.989) when comparing those with *TET2* mutations with those without. Among the patients treated with hypomethylating agents (HMAs) or hematopoietic stem cell transplantation (HSCT), the pooled HR for OS was 1.02 (95% CI: 0.77-1.35, p=0.89) and 1.54 (95%CI: 0.69 to 3.44; p=0.29), respectively. We also conducted an analysis of the response rate to HMAs, and the OR was 1.73 (95%CI: 1.11 to 2.70; p=0.016). Additionally, subgroup analyses showed the pooled HR for OS was 0.93(95%CI: 0.44 to 1.98; P=0.849) in WHO-classified CMML patients and 1.02(95%CI: 1.02 to 3.46; p=0.042) in studies evaluated *TET2* expression level. The analysis suggested *TET2* mutations had no significant prognostic value on MDS. However, the response rates to HMAs were significantly different between those with and without *TET2* mutations, and the low expression level of *TET2* gene was significantly associated with a poor OS in MDS patients.

## INTRODUCTION

Myelodysplastic syndromes (MDS) are a group of heterogeneous clonal diseases that originate in hematopoietic stem cells and are characterized by ineffective hematopoiesis and a high risk of transformation to acute leukemia [1–3]. The current prognostic scoring systems for patients with MDS are mainly based on karyotype abnormalities [4]. Recently, with the development of next-generation sequencing, it has become possible to identify new genomic aberrations. Increasing number of genomic aberrations have been reported to contribute to the development, progression and prognosis of myeloid neoplasms [5, 6]. Additionally, with the advances in therapeutic methods in MDS, some novel genomic aberrations have been reported to predict the effectiveness of specific treatment. Hence, genomic aberrations may offer more precise cancer phenotypes and more accurate estimations of the prognosis of MDS patients. Among these, the *TET2* gene is one of the most frequently mutated genes in MDS and CMML. However, the prognostic significance of *TET2* aberrations in MDS remains unclear. Although chronic myelomonocytic leukemia (CMML) has been eliminated from the MDS category by WHO classification, the WHO classification does not have any significant changes in the criteria for diagnosing CMML and most clinical studies still divide patients according to the FAB suggestions [7]. Therefore, in this report, we focus on the influence of TET2 mutations have on clinical prognosis of MDS and CMML patients. The *TET2* gene is a candidate tumor suppressor gene that resides at chromosome 4q24, and encodes a protein that catalyzes the conversion of the modified DNA base 5-methylcytosine (5mc) to 5-hydroxymethytosine [8–10]. Mutations in *TET2* gene were first identified in myeloid neoplasm, and they have been reported in 19-26% of MDS cases and 50% of CMML cases [11–19]. However, the precise role of *TET2* mutations in the prognosis of MDS patients remains controversial. O.Kosmider *et al* (2009b) [16] reported on *TET2* mutations in 88 patients with MDS and found that *TET2* mutations are an independent favorable prognostic factor in MDS. O.Kosmider *et al* (2009a) [15] suggested that *TET2* mutations were frequent adverse events in CMML. SMITH *et al* (2010) [12] reported that *TET2* mutations had no prognostic value on patients with MDS and CMML and Kim *et al* (2015) [20] indicated that *TET2* mutations were poor prognostic factor in patients with MDS. Hence, to gain full insight into the prognostic value of *TET2* mutations in patients with MDS, we performed this meta-analysis.

## RESULTS

### Study characteristics

**Figure 1:**
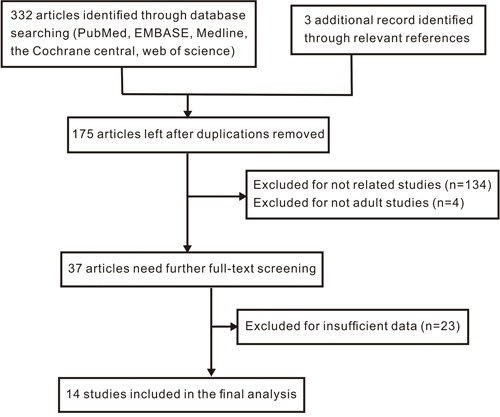
Flow diagram of selection process in the meta-analysis

As shown in Figure 1, 332 records were obtained by a systematic literature search. Three records were identified through relevant references. After excluding of 160 duplicates, 175 records remained for further screening. By reading the titles and reviewing abstracts, we excluded unrelated studies (n=134) and studies that were not performed on adults (n=4). Thus, 37 records remained for full-text screening. After carefully reading the full texts, 23 studies were eliminated due to insufficient data. Ultimately, 14 studies [12, 15, 16, 20–30] including an article [29] and a letter to the editor [20] were obtained and included in the meta-analysis.

### Characteristics of the included studies

Fourteen studies covering a total of 1983 patients were included in the meta-analysis. The characteristics are listed in Table 1. The 14 included studies were published between 2009 and 2015and included 395 *TET2* mutations and 141 *TET2* low gene expression level cases. All 14 eligible studies were retrospective studies. The sample size ranged from 39 to 439 and the frequency of *TET2* mutations in the included articles varied between 12.64 and 50.0%. After excluding studies that focused only on CMML patients, the frequency of *TET2* mutations ranged from12.64% to 27.23%. The results were similar to those of studies [13, 14, 17, 19]. Scopim-Ribeiro *et al* (2015) [22] included patients with both de novo AML and MDS, however, only patients with MDS were included in the meta-analysis. The median age in the eight studies [12, 15, 16, 22, 23, 25–27] was older than 60 years old, the median age was younger than 60 years old in four studies [20, 21, 24, 28] and the median age was not available in the other two studies [29, 30]. Patients in 10 eligible studies [12, 14, 20–27] were classified by WHO criteria, patients in 3 studies [28–30] were classified by FAB criteria and patients in KOSMIIDER et al (2009) [16] were classified by both WHO and FAB criteria. Patients in 2 studies [27, 30] were treated with HMAs, patients in two other studies [12, 16] were treated with either chemotherapy or transplant or supportive treatment, patients in two further studies [26, 28] were treated with stem-cell transplant, patients in Kim *et al* (2015) [20] were treated with either HMT or SCT or intensive chemotherapy, and patients in two studies [22, 23] did not receive any treatment, the treatment description was not available in some studies [15, 21, 24, 25, 29]. The median overall score of NOS results of the included studies was 7.5 (range 6-9), which indicated that the methodological quality was high (Table 2).

**Table 1:** Summary of the data extracted from the 12 studies included

Study	BEJAR *et al* (2014)[29]	KOSMIDER *et al* (2009)[16]	BRAUN *et al* (2011)[30]	SMITH *et al* (2010)[17]	X.Liu *et al* (2013)[31]	Kohlmann *et al* (2010)[32]	J. Wang et al (2012)[33]	O.Kosmlder *et al* (2009)[12]	Bejai *et al* (2011)[34]	Kim *et al* (2015)[20]	Christopeit *et al* (2015)[35]	Bejar *et al* (2014)[36]	Santamaria *et al* (2012)[37]	Scopim-Ribeiro *et al* (2014)[38]
Journal	*Blood*	*Blood*	*Blood*	*Blood*	*Leukemia & Lymphoma*	*J Clin Oncol*	*Leukemia Research*	Haematologica	N Engl J Med	Bone Marrow Transplantation	European Journal of Haematology	*J Clin Oncol*	Ann Hematol	European Journal of Haematology
Patients(n)	213	96	39	355	61	81	153	88	439	52	62	87	193	64
*TET2* status	58 mutations	22 mutations	13 mutations	55 mutations	10 mutations	36 mutations	35 mutations	44 mutations	90 mutations	8 mutations	13 mutations	11 mutations	96 low gene expression level	45 low gene expression level
Age(years)	≥70y(103)	71.5(63.5-79)	71(54-88)	64.4(17.0-96.)	58(23-80)	72.8(40-85.5)	51(16-81)	76(54-93)	≥75y(123)	52(18-73)	71(20-90)	58(19-73)	76(31-91)	68(16-90)
Criterion	FAB	FAB /WHO	WHO	WHO	WHO	WHO	WHO	WHO	FAB	WHO	WHO	FAB	WHO	WHO
Therapy	AZA alone(42) DEC alone(144) DEC+other(27)	None(40) Red blood cell transfusions(26) Erythropoiesis-stimulating agent with or without G-CSF(33) Lenalidomide/thalidomide(5) Demethylating agents(5) Low doses or intensive chemotherapy(7)	DAC(39)	None/BSC(211) EPO/GCSF(29) 5-azacitidine(14) Intensive chemo and transplant(82) Treatment data not available(19)	NR	NR	NR	NR	NR	HMT(43) SCT(8) Intensive chemotherapy(1)	Allogeneic hematopoietic stem cell transplantation(62)	Stem-Cell Transplantation(87)	Without any treatment	Without any treatment

WHO: World Health Organization; FAB: French-American-British; *TET2: Tet methylcytosine dioxygenase 2.* NR: not report

**Table 2:** Quality assessment of individual study

Study	Selection	Comparability	Outcome	Score
BEJAR *et al* (2014) [29]	****	**	**	8/9
KOSMIDER *et al* (2009) [16]	****	*	**	7/9
BRAUN *et al* (2011) [30]	****	*	***	8/9
SMITH *et al* (2010) [17]	****	*	***	8/9
X.Liu *et al* (2013) [31]	****	**	***	9/9
Kohlmann *et al* (2010) [32]	****		***	7/9
J. Wang *et al* (2012) [33]	****		***	7/9
O.Kosmlder *et al* (2009) [12]	****		***	7/9
Bejai *et al* (2011) [34]	****		***	7/9
Kim *et al* (2015) [20]	****		**	6/9
Christopeit *et al* (2015) [35]	***	**	***	8/9
Bejar *et al* (2014) [36]	****	**	**	8/9
Santamaria *et al*(2012) [37]	****		***	7/9
Scopim-Ribeiro *et al* (2014) [38]	****	**	**	8/9

(Selection: Representativeness of exposed cohort, Selection of no exposed cohort, Ascertainment of exposure, Outcome not present at start; Outcome: Assessment of outcome, Follow-up length, Follow-up adequacy).

Newcastle-Ottawa Quality Assessment Scale: study can have one star (*) for meeting each criterion, except that comparability (design or analysis) can have a maximum of two stars (**).

### Prognostic impact of TET2 mutations in patients with MDS

**Figure 2:**
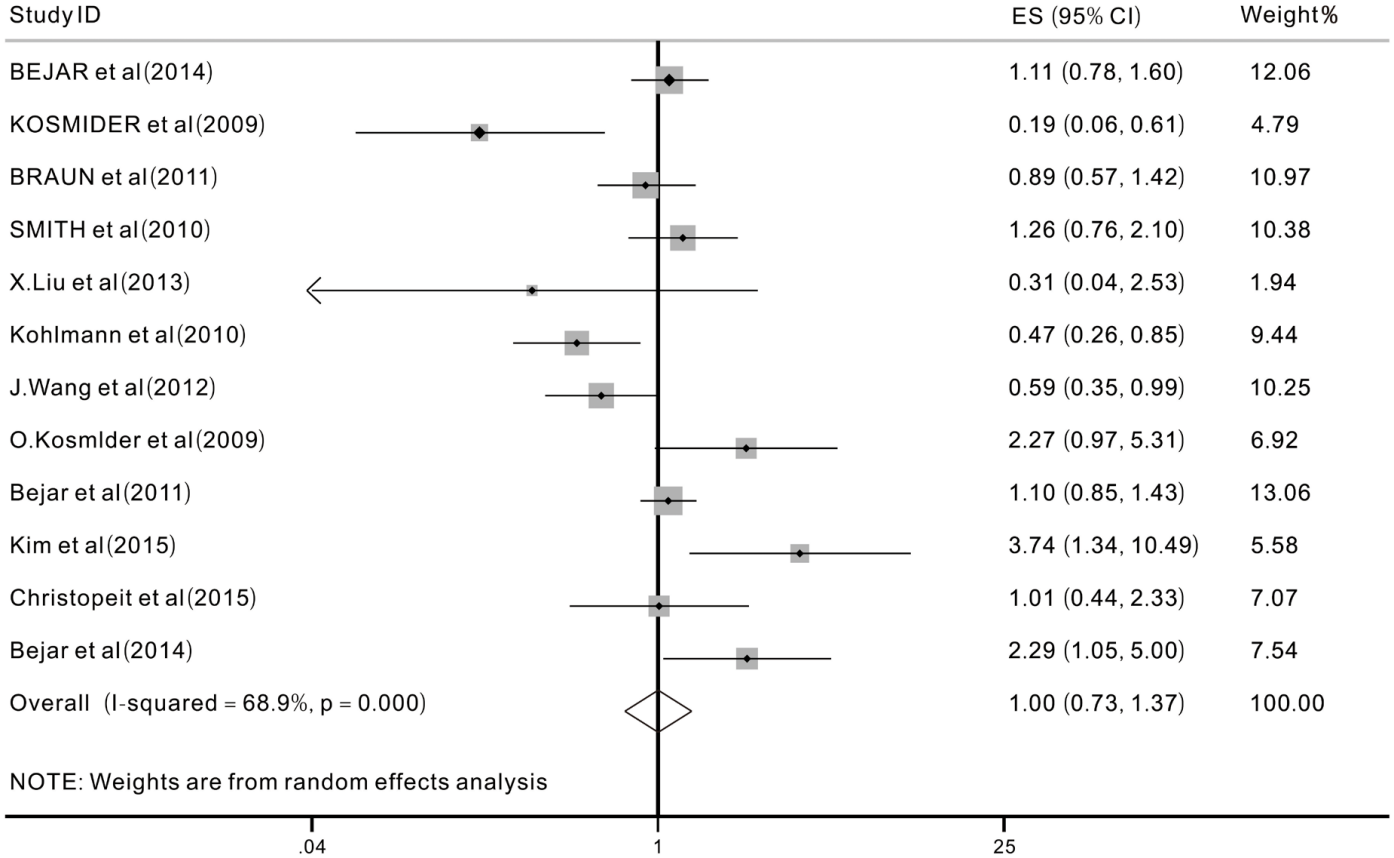
Forest plots of the hazard ratios (HRs) and 95% confidence intervals for overall survival (OS) in MDS patients The size of the blocks or diamonds represents the weight, the length of the straight line represents the width of 95% CI.

**Figure 3:**
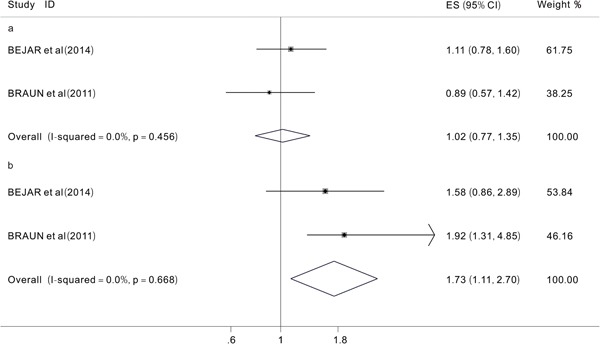
Forest plots of the hazard ratios (HRs) and 95% confidence intervals for overall survival (OS) in MDS patients treated with HMAs (a) and the hazard ratios (ORs) and 95% confidence intervals for response rates in MDS patients treated with HMAs (b) The size of the blocks or diamonds represents the weight, the length of the straight line represents the width of 95% CI.

**Figure 4:**
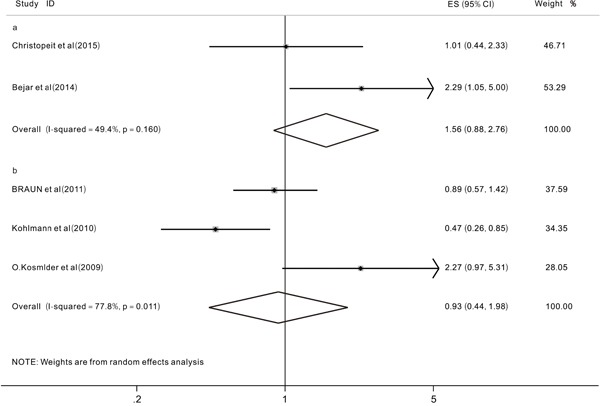
Forest plots of the hazard ratios (HRs) and 95% confidence intervals for overall survival (OS) in MDS patients treated with HSCT (a) and the hazard ratios (HRs) and 95% confidence intervals for overall survival (OS) in WHO-classified CMML patients (b) The size of the blocks or diamonds represents the weight, the length of the straight line represents the width of 95% CI.

We aimed to analyze two primary end points (OS and EFS) to investigate the prognostic impact of *TET2* mutations on MDS patients. However, after extracting useful data from the included studies, we were only able to analyze the prognostic impact of *TET2* mutations on the OS because of a lack of data on EFS. We evaluated 12 studies [12, 15, 16, 20, 21, 24–30] with a total of 1726 patients. The overall HR for the OS was 1.00 (95% CI: 0.74-1.37 with a p-value of 0.989, I^2^=68.9%) in MDS patients with *TET2* mutations compared to those without (Figure 2). These data indicated that the *TET2* mutations did not significantly affect the OS in patients with MDS. Then, different subgroup analyses were also performed. The results of one subgroup analysis showed that in patients treated with HMAs [27, 30], the pooled HR for the OS was 1.02 (95% CI: 0.77-1.35, p=0.89, I^2^=0.0%) (Figure 3a). Also, in this subgroup, we analyzed the response rate to treatment with HMAs between patients with and without *TET2* mutations, the results showed that when comparing patients with and without *TET2* mutations, the pooled OR for the response rate was 1.73 (95%CI: 1.11-2.7, P=0.016, I^2^=0.0%), which suggested that *TET2* mutations might predict the of response to HMAs in patients with MDS patients (Figure 3b). Another subgroup analysis was performed in MDS patients who had been treated with hematopoietic stem-cell transplant (HSCT). The pooled HR for the OS was 1.56 (95%CI: 0.88-2.76, p=0.125, I2=49.4%) when comparing patients with TET2 mutations to those without (Figure 4a) In addition, a subgroup analysis was conducted in the WHO classified CMML patients, the pooled HR for OS was 0.93 (95% CI: 0.44-1.98, P=0.849, I^2^=77.8%) (Figure 4b). The data indicated that *TET2* mutations did not significantly affect the OS in WHO classified CMML patients.

### Prognostic impact of TET2 expression levels in patients with MDS

**Figure 5:**
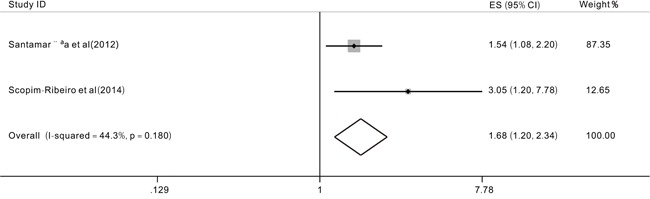
Forest plots of the hazard ratios (HRs) and 95% confidence intervals for overall survival (OS) according to the TET2 expression levels of patients with MDS patients The size of the blocks or diamonds represents the weight, the length of the straight line represents the width of 95% CI.

Studies [22, 23] focused on the role of the *TET2* expression level on the prognosis of MDS patients. The pooled HR for the OS was 1.68 (95% CI: 1.20-2.34, P=0.002, I2=44.3%) in the comparison of TET2 low expression with TET2 high expression (Figure 5). Because of a lack of data, we could not analyze the effect of the *TET2* expression level on the EFS. However, Santamarria et al (2012) [23] showed that the treatment-free survival (TFS, P<0.001) and progression-free survival (p=0.001) were shorter in the TET2 low expression level group than in the TET2 high expression level group. The results of Scopim-Ribeiro *et al* (2015) [22] suggested that a reduced *TET2* high expression negatively impacted the event-free survival (EFS, P≤0.05).

### Sensitivity analysis and publication bias

**Figure 6:**
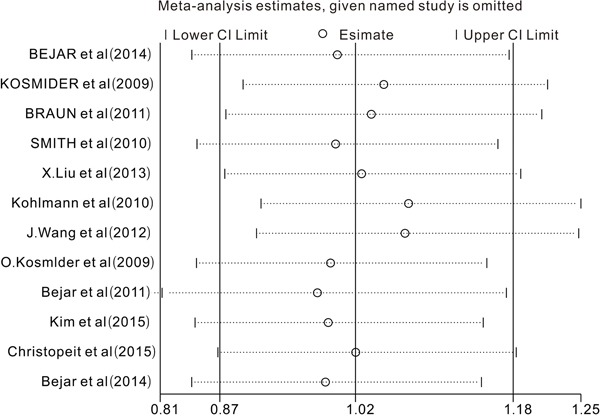
Sensitivity analysis The middle vertical axis represents the pooled HR and the 2 vertical axes indicate the corresponding 95% CI. Each hollow circle represents the pooled HR when the left study was omitted in this meta-analysis, and the 2 ends of every broken line indicate the 95% CI.

We conducted a sensitivity analysis by omitting one study at a time to assess the effect of the study quality on the stability of this meta-analysis. Only studies analyzing the role of *TET2* mutations on the prognosis were included in the sensitivity analysis. As shown in Figure 6, no individual study had a predominant influence on the overall HR. The Begg plots were largely symmetric, which indicated there was no evidence for significant publication bias in this meta-analysis.

**Figure 7:**
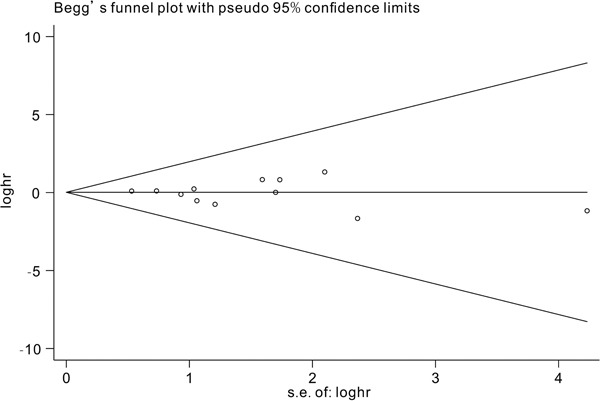
Begg's funnel plot for publication bias analysis Each point represents a separate study and horizontal line represents the mean effect size. Studies were distributed symmetrically and suggested there were no significant biases exist.

## DISCUSSION

*TET2* is an epigenetic enzyme that is capable of converting DNA 5-methylcytosine (5mc) to 5-hydroxymethylcytosine (5hmc). *TET2* mutations are a common event in a spectrum of myeloid malignancies and are one of the most frequent gene mutations in MDS and CMML [14, 31]. *TET2* mutations compromise the hydroxymethyl-catalytic activity of the epigenetic enzyme and can lead to low levels of 5-hmc in genomic DNA. This is thought to be one of the mechanisms through which *TET2* mutations contribute to the pathogenesis of MDS and CMML. Additionally, Wang *et al* [32] reported that *TET2* with *WT1* and *IDH1/IDH2* increased the 5-hmc levels. Although many studies have assessed the prognostic implication of *TET2* mutations in patients with MDS, the results are inconsistent and some are even conflicting [12, 15, 16, 20, 21, 24–30]. O.Kosmider *et al* (2009) [16] showed that *TET2* mutations were an independent favorable prognostic factor in MDS and were frequent and adverse events in CMML [15]. SMITH *et al* (2010) [12] reported that *TET2* mutations had no prognostic value for patients with MDS and CMML and Kim *et al* (2015) [20] indicated that *TET2* mutations were a poor prognostic factor in patients with MDS. As a result, the exact implication of *TET2* mutations on MDS still needed illuminating. Therefore, we performed a meta-analysis to exactly delineate the prognostic role of *TET2* mutations in MDS and CMML patients.

In this meta-analysis, 14 studies covering a total of 1983 patients were included. The results were as follows: the overall HR for the OS was 1.00 (95% CI: 0.74-1.37), which indicated that the *TET2* mutations did not significantly affect the OS in patients with MDS. Although the heterogeneity was large (I^2^=68.9%), the sensitivity analysis indicated the stability of our analysis, which demonstrated that the results of the meta-analysis were reliable. Our results are consistent with SMITH *et al* (2010) [12] and some other studies. Then, different subgroup analyses were conducted. The pooled HR for the OS was 0.93 (95% CI: 0.44-1.98) in WHO classified CMML patients. The results demonstrated that *TET2* mutations had no significant impact on OS in patients with CMML. This is consistent with the findings by BRAUN et al (2011) [27], but it is inconsistent with the reports by Kohlmann et al (2010) [25] and O.Kosmlder et al (2009) [15]. Although our results integrated data from multiple studies [22, 25, 30], which could be more reliable, the heterogeneity of out meta-analysis was extreme (I^2^=77.8%) and the number of studies included in the analysis was limited.

DNA HMAs are very effective drugs that have been approved for treating patients with MDS and HSCT is the only potentially curative therapeutic option in patients with MDS. Hence, we also performed subgroup analyses in patients who were treated with HMAs and HSCT, The results showed that the pooled HR for the OS was 1.02 (95% CI: 0.77-1.35) and the pooled OR for the response rate was 1.73 (95% CI: 1.11-2.70) for patients who were treated with HMAs. Although a randomized phase 3 study by Fenaux *et al* (2009) [33] indicated that HMAs conferred an overall survival benefit compared with supportive care, HMAs did not prolong the OS of MDS patients with *TET2* mutations compared to those without in studies [27, 30]. Our results were consistent with the results of a couple studies [27, 30]. Additionally, our results showed that MDS patients with TET2 mutations had an increased response rates to HMAs compared with WT and this is consistent with many prior studies [27, 30]. Additionally, in patients who received HSCT, we found that *TET2* mutations had no significant impact on the OS. However, we only included two studies in this subgroup analysis, and the exact role of TET2 mutations in MDS patients treated with HSCT requires further investigation.

It has been reported that *TET2* haploinsufficiency may be sufficient to confer myeloid transformation and impair hematopoietic cell differentiation. Hence, we conducted a meta-analysis to investigate the *TET2* mRNA expression. Two studies were included in the analysis. The pooled HR for the OS was 1.68 (95%CI: 1.20-2.34) when comparing *TET2* low expression level with TET2 high expression. The results indicated that TET2 low expression was associated with an unfavorable prognosis in MDS patients. This is consistent with prior studies [22, 23]. However, there are three main limitations in our meta-analysis. First, the analysis was based on observational studies rather than on randomized trials or prospective studies. Second, the analysis, especially the subgroup analysis covered a small number of MDS patients. Third, we could not avoid potential heterogeneity and publication bias in the meta-analysis.

## CONCLUSION

Considering the limitations mentioned above, our meta-analysis shows that *TET2* mutations have no significant impact on the OS of MDS patients. Additionally, in WHO- classified CMML patients and patients treated with HMAs or stem-cell transplantation subgroups, *TET2* mutations did not have prognostic value. However, patients with *TET2* mutations achieved higher response rates to HMAs than did those without mutations and a low *TET2* expression level in patients with MDS was significantly associated with a poor OS. The results suggest that *TET2* mutations may be predictive of the response to HMAs in patients with MDS and *TET2 gene* expression level may provide additional information for a suitable molecular risk-stratification in MDS.

## MATERIALS AND METHODS

### Search strategy

The search for eligible studies was conducted in PubMed, Embase, Medline, the Cochrane Library and Web of Science with the following search terms: *“TET2”* or *“tet methylcytosine dioxygenase 2”* or *“tet oncogene family member 2”* and “MDS” or “myelodysplastic syndrome” or “myelodysplasia” or “preleukemia” or “CMML” or “Chronic myelomonocytic leukemia”. The search was restricted to human studies, and free articles with no language limitation. Relevant papers published between 2007 and 2016 were obtained by two independent reviewers (Y.L and Z.J.). We also reviewed the references for missing information.

### Selection criteria

We included trials if they met the following criteria: (1) published between January 01, 2007 and July 31, 2016 as original articles; (2) assessed the association between the *TET2* status and prognosis in MDS and CMML patients; and (3) offered detailed survival information from which we could calculate the hazard ratios (HRs) as well as the corresponding 95% confidential intervals (CIs) or event-free survival (EFS) based on the following *TET2* status: *TET2 mutations/wild-type TET2 or TET2 low expression level/TET2 high expression*. The OS was measured from the date of the first sample collection to the time of death from any causes or to the time at the last follow-up (censored). The EFS was defined as remission, induction failure, relapse or death for any reason from entry in the trial. We excluded studies that were published in the abstract form, review articles, case reports, only analyzing pediatric patients or studies with unavailable or incomplete data. However, we included a letter to the editor in our meta-analysis because it met all of the inclusion criteria.

### Data extraction

Two reviewers (Y.L and Z.L.) independently extracted data from the articles. Disagreements were resolved by discussion until a consensus was achieved. The following data were extracted from the articles: the name of the first author, year of publication, journal, number of patients, number of *TET2* mutations/*TET2* low expression level, age, criteria for classification of MDS and CMML, therapeutic method and outcomes such as the including hazard ratios (HRs) or response ratios, and their 95% confidence intervals (CIs) for the OS based on the *TET2* status. When outcomes published in the original articles were only survival curves, the HRs and 95% CIs were calculated by the methods proposed by Parmar *et al* (1998) [34] and Hotta *et al* (2004) [35]. If HRs of the univariate analysis and multivariate analyses were reported, the results of the multivariate analysis including other variables should be preferentially considered because they could be more accurate.

### Quality assessment

Two reviewers (Y.L and Z.L.) independently assessed the study quality. The Newcastle-Ottawa (NOS) [36] was used to score the quality of each cohort study. This scale has nine items that are classified into the following three major categories: selection (four items), comparability (two items) and outcome (three items). The overall quality score was classified the following into 3 types: high quality (7-9 scores), intermediate quality (4-6 scores), and low quality (1-3 scores). Any discrepancies were resolved among the authors.

### Statistical analysis

All statistical analyses were performed using Stata ver.12 software (College Station, TX, USA). For the OS and response rate, the HRs or ORs and their 95% CIs were directly extracted from the included studies or indirectly calculated from the reported events, the P value in the log-rank test or from the published Kaplan-Meier curves [37–39]. The prognostic role of *TET2* mutations on the OS and response ratio were assessed by estimation of the pooled HRs and the respective 95% CIs with the inverse variance method in total population and subgroups [40]. The statistical heterogeneity of the studies was assessed by the chi-square based Q-test and quantified with the I^2^ statistic (I^2^=0-25%; no heterogeneity; I^2^=25-50%; moderate heterogeneity; I^2^=50-75%; large heterogeneity; and I^2^=75-100%; extreme heterogeneity). When the heterogeneity across studies was identified (>50%), the random effects model (the DerSimonian and Laird method) was used, otherwise, the fixed-effect model (the Mantel-Haenszel method) was used [41]. Additionally, sensitivity analysis was conducted to investigate the influence of one single study on the overall HR and Begg's and Egger's tests were conducted to detect possible publication bias [40, 41]. A two-tailed P-value less than 0.05 was considered statistically significant. All the statistical analyses were done by Y.L and K.C.
